# Incomplete denitrifying bacteria drive N_2_O fluxes in ancient Siberian permafrost microcosms

**DOI:** 10.1093/femsec/fiag034

**Published:** 2026-03-31

**Authors:** Yanchen Sun, Xiaofen Wu, Oksana G Zanina, Elizaveta M Rivkina, Karen G Lloyd, Frank E Löffler, Tatiana A Vishnivetskaya

**Affiliations:** Department of Civil and Environmental Engineering, University of Tennessee, Knoxville, TN 37996, United States; Center for Environmental Biotechnology, University of Tennessee, Knoxville, TN 37996, United States; Kovda Institute of Physicochemical and Biological Problems in Soil Science, Russian Academy of Sciences, Pushchino 142290, Russia; Kovda Institute of Physicochemical and Biological Problems in Soil Science, Russian Academy of Sciences, Pushchino 142290, Russia; Department of Microbiology, University of Tennessee, Knoxville, TN 37996, United States; Department of Civil and Environmental Engineering, University of Tennessee, Knoxville, TN 37996, United States; Center for Environmental Biotechnology, University of Tennessee, Knoxville, TN 37996, United States; Department of Biosystems Engineering and Soil Science, University of Tennessee, Knoxville, TN 37996, United States; Department of Biochemistry & Cellular and Molecular Biology, University of Tennessee, Knoxville, TN 37996, United States; Center for Environmental Biotechnology, University of Tennessee, Knoxville, TN 37996, United States; Department of Microbiology, University of Tennessee, Knoxville, TN 37996, United States

**Keywords:** nitrous oxide, thawing permafrost, greenhouse gas, incomplete denitrifying N_2_O reducers, arctic ecosystems

## Abstract

Nitrous oxide (N_2_O) contributes to stratospheric ozone depletion and global warming. Knowledge about microbial formation and consumption of N_2_O in old permafrost remains limited. Permafrost samples collected on the East Siberian Sea coast of Russia from a single borehole at depths of 5.4 and 16.9 m, which showed presence of nitrogen substances and nitrogen cycling genes, were used to establish microcosms supplemented with NO_3_^−^ and N_2_O to assess denitrification and N_2_O consumption at 4°C and 20°C. Rapid N_2_O formation was observed in NO_3_^−^-supplemented microcosms, but N_2_O consumption was slow and incomplete over a 1-year incubation in all microcosms. Twenty-three qualified metagenome-assembled genomes (MAGs) harboring genes involved in NO_3_^−^ and/or N_2_O reduction were recovered from both NO_3_^−^- and N_2_O-supplemented microcosms. Twenty MAGs represent novel taxa. Four MAGs, two of each from NO_3_^−^- and N_2_O-supplemented microcosms, contained *nosZ* genes indicating N_2_O consumption potential, however the complete denitrification (i.e. NO_3_^−^→N_2_) gene sets were not detected in these MAGs. Though, N_2_O production exceeded N_2_O consumption in NO_3_^−^-supplemented microcosms at 4°C. Our microcosm experiments suggest N_2_O formation surpasses its consumption in newly thawed ∼120 kyr old permafrost, emphasizing the importance of using integrated approaches to assess and predict N turnover in response to permafrost degradation.

## Teaser

Thawing ancient Siberian permafrost in NO_3_^-^ and N_2_O-supplemented microcosms led to N_2_O accumulation, and MAGs indicated the emissions were driven by incomplete denitrifying bacteria.

## Introduction

Perennially frozen sediments, deposits, or soils are called permafrost and cover ∼17% of the global terrestrial surface (Gruber 2012), preserving about 50% of the total global soil organic carbon and nitrogen (Batjes 1996, Harden et al. 2012, Drake et al. 2015). Global warming accelerates thawing of permafrost, releasing a large amount of greenhouse gases [i.e. carbon dioxide (CO_2_), methane (CH_4_), and nitrous oxide (N_2_O)] exacerbating climate change (i.e. positive feedback loop) (Schuur et al. 2009, Voigt et al. 2017, Knoblauch et al. 2018, Voigt et al. 2020, Lacroix et al. 2022, Waldrop et al. 2025). Under the current scenario, the temperature in Artic regions would increase by 5.6°C–12.4°C by the end of 2100 (Christensen et al. 2013), which is predicted to lead to extensive thawing and permafrost degradation (Lawrence et al. 2012). Thawing permafrost is a potentially significant source of greenhouse gases in the coming decades and centuries, but our understanding of the microbiology driving these processes is limited.

N_2_O is a relevant greenhouse gas that has been underappreciated in traditional global greenhouse gas budgets in the Arctic (Voigt et al. 2017, 2020, Marushchak et al. 2021, Lacroix et al. 2022). N_2_O has higher global warming potentials than the equivalent amounts of CO_2_ and CH_4_ (IPCC 1996, Ravishankara et al. 2009, Etminan et al. 2016). Permafrost thawing causes the decomposition of soil organic nitrogen, ultimately leading to continued emission of N_2_O (Elberling et al. 2010, Voigt et al. 2017, 2020, Marushchak et al. 2021). For instance, N_2_O emissions reaching 3 mg and 0.9 mg N_2_O m^−2^ d^−1^ have been reported from thawing peatland (Voigt et al. 2017) and Yedoma permafrost (Marushchak et al. 2021), respectively, with an average of 0.29 mg N_2_O m^−2^ d^−1^ across all permafrost types (Voigt et al. 2020). These measurements suggest that permafrost-affected soils are N_2_O emitters and N_2_O sources outpace N_2_O sinks. The overall emissions of N_2_O are collectively determined by formation versus consumption (i.e. N_2_O fluxes). Microbial processes control N_2_O formation and consumption, and a comprehensive understanding of the microbiology in thawing permafrost is necessary to predict future emission scenarios.

Microbial formation of N_2_O results from multiple processes, including denitrification (NO_3_^−^/NO_2_^−^→N_2_O) (Knowles 1982), nitrification (NH_4_^+^→NO_3_^−^) (Wrage et al. 2001), dissimilatory nitrate reduction to ammonium (DNRA) (NO_3_^−^/NO_2_^−^→NH_4_^+^) (Tiedje et al. 1983), and chemodenitrification (i.e. the ferrous iron-mediated abiotic reduction of NO_2_^−^ to N_2_O) (Chalk and Smith 1983, Onley et al. 2018). In contrast to the diverse sources of N_2_O, the major biological sink of N_2_O is its reduction catalyzed by N_2_O reductase (NosZ) (Sanford et al. 2012). Importantly, the two canonical *nosZ* clades (Clade I and Clade II) have distinct biochemical properties and occur in different ecological niches, therefore a comprehensive understanding of each of these clades is crucial for predicting microbial responses to climate change and potentially managing N_2_O emissions (Yoon et al. 2016, Hallin et al. 2018). Studies of N_2_O emissions from thawed permafrost and active layer soils are mainly conducted by measuring N_2_O fluxes in field or mesocosms experiments (Elberling et al. 2010, Voigt et al. 2017, Marushchak et al. 2021); however, the relative contributions of N_2_O formation versus consumption to the overall N_2_O flux, as well as microorganisms involved in these two processes, have not been fully elucidated in pristine ancient permafrost.

N_2_O emissions from Arctic thawing permafrost have been documented (Voigt et al. 2017, Marushchak et al. 2021). A recent study demonstrated that N_2_O emissions are affected by underlying permafrost type, with seasonally thawed active layers releasing more N_2_O than freshly thawed permafrost (Marushchak et al. 2021). This observation may be due to microbial community responses to freeze-thaw cycles (Monteux et al. 2020, Abramov et al. 2021). Microbial communities in the top active layer (i.e. seasonally-thawed region) could migrate to deeper active layers (Tecon and Or 2017) to reach the so-called permafrost table that prevents further penetration into deeper, permanently frozen layers. Such physical effects unique to permafrost soils complicate investigations of permafrost microbial communities involved in N_2_O formation and consumption in their natural habitat.

Permafrost soils collected from the East Siberian Sea coast of Russia offer a unique opportunity to study soils that have not experienced any freeze-thaw events and are therefore free of any exposure from microbial migration from upper layers (Liang et al. 2021, Wu et al. 2023). Using these samples offers opportunities for laboratory exploration that more closely mimics natural thawing scenarios, since a warming climate is expected to impact deeply buried permafrost that has not been influenced by microbial migration from upper layers. Our previous comparative analysis of metagenomes from these pristine Siberian permafrost samples revealed the presence of nitrogen cycling genes in samples where NO_3_^−^, NO_2_^−^, and NH_4_^+^ were detected (Wu et al. 2023). These permafrost horizons, having experienced no reported freeze-thaw cycles for thousands of years, provide an excellent system for investigating microorganisms potentially implicated in N_2_O turnover. Different nitrogen species (e.g. NO_3_^−^, NO_2_^−^, NH_4_^+^) were detected in these coastal permafrost deposits (Janssen and Bock 1994). Therefore, we hypothesized that microbial communities preserved in the Siberian Sea coastal permafrost have the metabolic potential for both N_2_O formation and consumption.

The objectives of this study were to examine N_2_O formation and consumption potential exhibited after abrupt thawing of ancient Siberian permafrost soils in laboratory microcosms and to identify key taxa driving these two processes. The production of N_2_O via denitrification and the consumption of N_2_O was monitored in a series of microcosms incubated for 1 year under different temperature conditions (4°C and 20°C). Responses of the permafrost microbial communities were assessed through geochemical measurements and metagenome analysis.

## Materials and methods

### Permafrost samples

Permafrost at the East Siberian Sea coast, the northern boundary of the Kolyma Lowland in northeastern Siberia, Russia, was formed about 105–120 kyr ago. In addition to being old, these permafrost deposits offer a depositional transition between two very different environments. The upper horizon (depth of 5.4 m) contains silty loam sediments that are representative of coastal brackish permafrost (designated 54BP) and the deeper layer (depth of 16.9 m) that are sandy saline silt loams of marine permafrost (designated 169MP). Cores were retrieved in August 2017 from a single borehole following an aseptic sampling protocol (Liang et al. 2019) and only the inner frozen portions of the core (∼5 cm in diameter) was subsampled and stored at −20°C. During the fieldwork in August 2017, air temperature fluctuated from 5°C to 23°C, the active layer temperature declined with depth from 6°C at 20 cm and 2°C at 40 cm to 0.5°C at the lower horizons of active layer, whereas the average temperature in the permafrost at 5.4 and 16.9 m was about −7° and −8°C, respectively. Continuous monitoring of permafrost temperature showed its stable increase at rate of 0.11°C per year (Ostroumov et al. 2021). For microcosms experiments, samples from depths of 5.4 and 16.9 m were selected because they differ in content of carbon, nitrogen, and salinity (Fig. S1) as described, and metagenome analysis showed the presence of denitrification genes (Wu et al. 2023). The physicochemical properties of the permafrost samples have been described (Table S1).

### Permafrost microcosms

Defined, bicarbonate-buffered (pH 7.2) mineral salt medium was prepared following established protocols (Sun et al. 2024). l-cysteine (0.2 mM) was added as a reductant. Microcosms were set up according to an established procedure (Sun et al. 2024). Briefly, 8 g of each frozen permafrost sample, 54BP and 169MP, were homogenized aseptically, and 1-g aliquots were transferred to 60-ml glass serum bottles containing 30 ml of medium using sterilized stainless-steel spatulas inside a glove box (Coy Laboratory Products, Grass Lake, MI, USA) filled with 97% N_2_ and 3% H_2_ (v: v). The serum bottles were immediately sealed with sterile butyl rubber stoppers, crimped with aluminum caps, removed from the glove box and kept inverted. Pyruvate (5 mM) was added using a 1-ml plastic syringe from an anoxic, filter-sterilized 1 M stock solution and additional feedings occurred after 6 and 12 months to supply sufficient electron donor. A total of 0.3 mmol of pyruvate was added to each microcosm over the 1-year incubation period. In addition, the Wolin vitamin mix and CuCl_2_ (∼17 μM) were added from concentrated stock solutions to individual bottles (Wolin et al. 1963). The bottles were incubated at 4°C and 20°C under static conditions. The choice of temperatures was based on field measurements and published data. The 4°C condition was selected to reflect typical summer temperatures in Arctic soil (Marushchak et al. 2021, Lacroix et al. 2022), while the 20°C condition was chosen to simulate an extreme warming event (Marushchak et al. 2021, Ostroumov et al. 2021, Lacroix et al. 2022).

**Figure 1:**
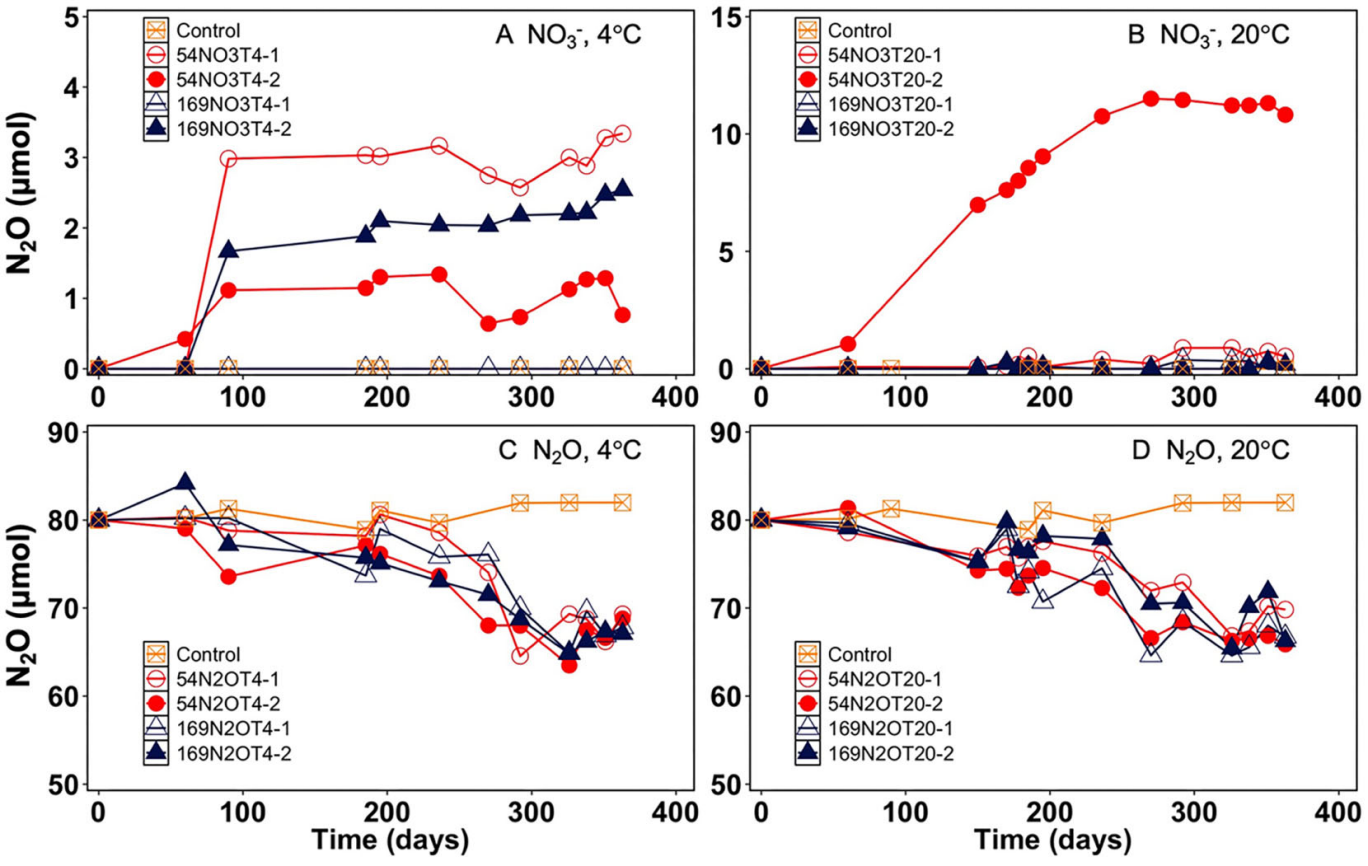
N_2_O profiles in permafrost soil microcosms. Panels A and B show N_2_O production in NO_3_^−^-supplemented microcosms incubated at 4°C and 20°C. Panels C and D show N_2_O consumption in N_2_O-supplemented microcosms at 4°C and 20°C. The red circles and blue triangles represent the permafrost samples from depths of 5.4 m and 16.9 m, respectively. The orange squares in panels C and D represent the heat-killed controls. Each type of microcosm has two replicates shown as open symbols for replicate 1 and closed symbols for replicate 2. Heat-killed controls represent single microcosms.

NO_3_^−^ and N_2_O (nominal concentration) were amended at 1 mM, consistent with concentrations commonly used in Arctic soil microcosms and denitrification batch experiments to probe process potentials without strong substrate limitation (Herndon et al. 2015, Song et al. 2022, Zhang et al. 2023). Plastic syringes with 25-gauge needles (BD, Franklin Lakes, NJ, USA) were used to add 1 mM NO_3_^−^ (30 µmol) from a 1 M stock solution or 1 mM N_2_O (2 ml or 80 µmol undiluted N_2_O). N_2_O was measured at irregular time intervals throughout the incubation using a gas chromatograph (GC) (details are described in “Analytical procedures” below) (Yin et al. 2018). NO_3_^−^ and NO_2_^−^ were analyzed using a uniform time intervals by ion chromatography (IC) in liquid samples (0.5 ml) withdrawn from the microcosms (Onley et al. 2018). Sampling frequency varied over the course of the incubation, with denser sampling during periods of rapid change, as reflected by the time points shown in Fig. 1.

The limited amount of permafrost materials available allowed the setup of 16 microcosms supplemented with either NO_3_^−^ or N_2_O and incubated at 4°C and 20°C. Microcosm names reflect sample depth, amendment, and incubation temperature; i.e. 54N2OT4, 54N2OT20, 54NO3T4, 54NO3T20, 169N2OT4, 169N2OT20, 169NO3T4, and 169NO3T20 (for example, 54N2OT4 refers to microcosms established with 5.4 m depth permafrost supplemented with N_2_O and incubated at 4°C; for further details on microcosm nomenclature, see Supplementary Materials). Duplicate microcosms per treatment were established. Negative controls included duplicate heat-killed (autoclaved) microcosms and microcosms that received 5 mM pyruvate but no N_2_O and NO_3_^−^.

### Analytical procedures

N_2_O was analyzed with an Agilent 3000A Micro-GC (Agilent, CA, USA) equipped with a thermal conductivity detector and a Plot Q column (Zhang et al. 2023, Sun et al. 2024). The injector and column temperatures were set to 100°C and 50°C, respectively, and the column pressure was 25 psi. A gas sample (0.1 ml) was withdrawn from each microcosm’s headspace and manually injected into the Micro-GC. Aqueous N_2_O concentrations were calculated from the headspace concentration using a dimensionless Henry’s constant for N_2_O of 1.68 and based on the equation *C*_aq_ = *C*_g_/*H*_cc_ (Sander 1999). *C*_aq_, *C*_g_, and *H*_cc_ are the aqueous N_2_O concentration (μM), the headspace N_2_O concentration (μM), and the dimensionless Henry’s constant, respectively. The total amount of N_2_O equaled the sum of N_2_O in the headspace and in the aqueous phase.

NO_3_^−^ and NO_2_^−^ were quantified by IC using a reagent-free eluent regeneration system (ICS-2100; Dionex, Sunnyvale, CA) and a Dionex IonPac AS18 4-mm by 250-mm analytical column heated to 30°C. The eluent was 10 mM KOH and the flow rate was 1 ml min^−1^. For each measurement, a 0.5 ml liquid sample was withdrawn from the microcosm, filtered through a 0.2 μm polyethersulfone membrane filter (Nalgene, Rochester, NY, USA), and the NO_3_^−^ and NO_2_^−^ concentrations were determined in the filtrate.

### DNA extraction and metagenome sequencing

At the end of the 1-year incubation period, the microcosms were sacrificed. Following vigorous shaking, ∼5 ml of homogenized slurry was removed with 5 ml plastic syringes equipped with 18-gauge needles, and total genomic DNA (gDNA) was extracted using the DNeasy PowerSoil kit (Qiagen, Hilden, Germany) following the manufacturer’s protocol. DNA concentrations were determined using the Qubit fluorometer (Life Technologies, Carlsbad, CA). Genomic DNA extracted from the pristine permafrost and microcosm slurries was used to prepare Illumina shotgun metagenome libraries with the Nextera DNA Flex Library Preparation Kit (Illumina, Inc., San Diego, CA) following the manufacturer’s instructions. The libraries were sequenced at the University of Tennessee Genomics Core using the Illumina NovaSeq 6000 platform, SP 500 reagent kit (Illumina, Inc., San Diego, CA) to generate paired-end reads (2 × 250 bp; Table S2).

### Bioinformatic analyses

The raw metagenomic reads derived from pristine permafrost 54BP and 169MP (Table S3) (Wu et al. 2023) and the 16 microcosms were trimmed using Trimmomatic v.0.39 with default settings (Bolger et al. 2014). Metagenomic data from two biological replicates of the same type of microcosm were co-assembled. Assembly of the trimmed short reads was performed using MEGAHIT v.1.2.9 (Li et al. 2015) and metaSPAdes v3.10.1 (Nurk et al. 2017), and contigs longer than 1000 bp in length were routed into downstream analyses after dereplicating with CD-HIT (Li and Godzik 2006). Contigs were binned using MaxBin2 v.2.2.4 (Wu et al. 2016), MetaBAT v.2.12.1 (Kang et al. 2019), and CONCOCT v.1.1.0 (Alneberg et al. 2014) with default settings, respectively, to recover individual metagenome-assembled genomes (MAGs). An optimized, non-redundant set of MAGs was recovered using metaWRAP with the command bin_refinement from MAGs generated by using three different binning packages (Uritskiy et al. 2018). The resulting MAGs were checked for completeness and contamination using CheckM v.1.0.18 (Parks et al. 2015). The coverage of each MAG was calculated using CoverM v0.6.1 (Aroney et al. 2025) after removing bases in the lowest and highest 5% of per-base coverage values to reduce the influence of mapping and assembly artifacts and obtain a robust estimate of average MAG coverage (Wu et al. 2021, 2023). For each metagenomic library (pristine permafrost and microcosm slurries), the relative MAG abundance was then defined as the fraction of quality-filtered reads mapping to a given MAG normalized by the total number of reads in that library, i.e. as relative read recruitment to each MAG (Pasolli et al. 2019). Because this metric does not explicitly correct for genome length and can bias absolute abundance estimates toward larger genomes, we interpret relative MAG abundances primarily as within-MAG differences across treatments rather than as quantitative comparisons among MAGs of different genome size.

### Microbial community profiling

GraftM v.0.13.1 was used to extract 16S rRNA gene fragments from the trimmed metagenomic datasets and to assign taxonomy using the default Greengenes 16S rRNA gene package (release 13_8), which is based on the 97% nucleotide identity representative reference set (McDonald et al. 2012, Boyd et al. 2018). Taxonomic classification was performed within GraftM via sequence placement onto a pre-constructed phylogenetic reference tree derived from the Greengenes database. Relative abundance was estimated from the number of reads/fragments assigned to each taxon, and community profiles were summarized at the phylum and family levels based on these taxonomic assignments.

### Functional annotation of assembled contigs

To provide more comprehensive information about nitrogen turnover potential, a functional analysis of the assembled contigs was performed. Briefly, protein-coding sequences present in assembled contigs were predicted using Prodigal v.2.6.3 (Hyatt et al. 2010), and assigned Kyoto Encyclopedia of Genes and Genomes (KEGG) orthologs (KOs) using KofamScan v.1.3.0 against hidden Markov model (HMM) profiles from the KEGG database (released on Aug-1–2024) (Aramaki et al. 2020). The completeness of various metabolic pathways was assessed using KEGG-Decoder v.1.32.0 using KOALA_definitions as the reference (Graham et al. 2018), with a specific focus on nitrogen cycling, copper transport, pyruvate, and hydrogen metabolism. In addition, quality-filtered reads from each sample were mapped to a curated set of *nosZ* reference sequences from the ROCker models database (http://enve-omics.ce.gatech.edu/rocker/models), which revealed that only very low numbers of reads mapped to *nosZ* (data not shown).

### Taxonomy, functional annotation, and comparative genomics

MAGs were assessed following minimum information about a MAG (MIMAG) criteria (Bowers et al. 2017). For downstream analyses, we retained MAGs defined as qualified with completeness >70% and contamination <10%. This set included MAGs that met the MIMAG high-quality requirements (>90% completeness, <5% contamination) and MAGs with completeness between 70%–90% from the medium-quality group (≥50% completeness, <10% contamination). Taxonomic assignments for each qualified MAG used the GTDB-Tk v.2.4.0 (Chaumeil et al. 2022) based on the Genome Taxonomy Database (GTDB, https://github.com/Ecogenomics/GTDBTk) taxonomy R220 (Parks et al. 2020). Average nucleotide identity (ANI) was used to assess taxonomic novelty of the MAGs on the basis of the 95% ANI threshold (Goris et al. 2007). Functional analysis of qualified MAGs was performed following the same steps as for contigs. MAGs encoding nitrogen cycling genes were collected based on the KEGG annotation results.

### NosZ phylogeny

NosZ reference sequences, including Clade I and Clade II nosZ, were compiled from the ROCker models database and used as a curated reference set for phylogenetic reconstruction (Sun et al. 2024). These sequences were aligned with ClustalΩ using default settings (Sievers et al. 2011). A maximum likelihood reference tree was built using the alignment of NosZ in RAxML V8.2.12 with ‘-f a’ algorithm (Stamatakis 2014). The translated sequences of near full-length NosZ derived from assemblies and MAGs were added to the NosZ reference protein alignment using ClustalΩ (Sievers et al. 2011), and the new alignment was placed in the NosZ reference phylogenetic tree using the RAxML EPA algorithm (-f v option). The generated jplace file was further processed using a script (available through http://enve-omics.ce.gatech.edu/) for visualization in iTOL (Letunic and Bork 2016).

### Statistics

Statistical analyses were performed using R v.4.0.2 (R Core Team 2021). Beta-diversity was calculated by incorporating both the presence/absence of taxa and their relative abundances using weighted-UniFrac distances and visualized using the principal coordinate analysis (PCoA) plot in R with packages ggplot2 (Gómez-Rubio 2017) and phyloseq (McMurdie and Holmes 2013). Statistical differences in microbial communities among pristine permafrost samples, microcosms incubated at 4°C, and microcosms incubated at 20°C were determined using permutational multivariate analysis of variance (PERMANOVA) with the adonis function in vegan with 999 permutations (Oksanen et al. 2013). KEGG annotation results of qualified MAGs were illustrated using heatmap in R with package pheatmap (Kolde and Kolde 2015).

## Results

### N_2_O formation and consumption in permafrost soil microcosms

NO_3_^−^ reduction was observed in all microcosms established with permafrost collected from 5.4 m depth and incubated at both 4°C and 20°C (Fig. S2 A and B); however, the consumption of all added NO_3_^−^ only occurred in microcosms 54NO3T4-1 (Fig. S2A) and 54NO3T20-2 (Fig. S2B). NO_3_^−^ reduction was not prevalent in permafrost from 16.9 m depth and only a single microcosm, 169NO3T4-2, showed NO_3_^−^ reduction at 4°C (Fig. S2A). After a 1-year incubation, the NO_3_^−^-supplemented microcosms yielded different amounts of NO_2_^−^ (Figs S2 C and D) and N_2_O (Fig. 1 A and B) depending on sample depth and incubation temperature. N_2_O production plateaued after 3 months of incubation with concentrations ranging between 1.4 and 3.2 μmol in all NO_3_^−^-supplemented microcosms incubated at 4°C (Fig. 1A). In contrast, NO_3_^−^ reduction at 20°C in the microcosm established with 5.4 m permafrost transiently produced up to 6.3 μmol NO_2_^−^ (Fig. S2D) and up to 11.5 μmol of N_2_O were produced (Fig. 1B). No N_2_O was detected in heat-killed control microcosms that did not receive NO_3_^−^, indicating that abiotic N_2_O formation from nitrogenous compounds associated with the permafrost, or the ammonium supplied with the medium, did not occur or was negligible.

Measurable N_2_O consumption occurred in all N_2_O-supplemented microcosms at 4°C (Fig. 1C) and at 20°C (Fig. 1D). Over a 1-year incubation period, the average N_2_O consumption at 4°C was 10.9 ± 0.4 and 12.6 ± 0.5 μmol in the microcosms established with 5.4-m depth and 16.9-m depth permafrost, respectively, with an average reduction rate of 0.032 ± 0.003 μmol day^−1^ g^−1^ (wet weight). At 20°C, slightly more N_2_O was consumed, 12.2 ± 2.8 and 13.5 ± 0.3 μmol in 5.4-m and 16.9-m depth microcosms, respectively, with an average reduction rate of 0.035 ± 0.005 μmol day^−1^ g^−1^ (wet weight). No measurable N_2_O consumption was detected in heat-killed control microcosms (Fig. 1 C and D). Evidently, thawing East Siberian Sea coast permafrost has the potential for microbial reduction of N_2_O.

### Microbial community composition based on 16S rRNA gene analyses

**Figure 2:**
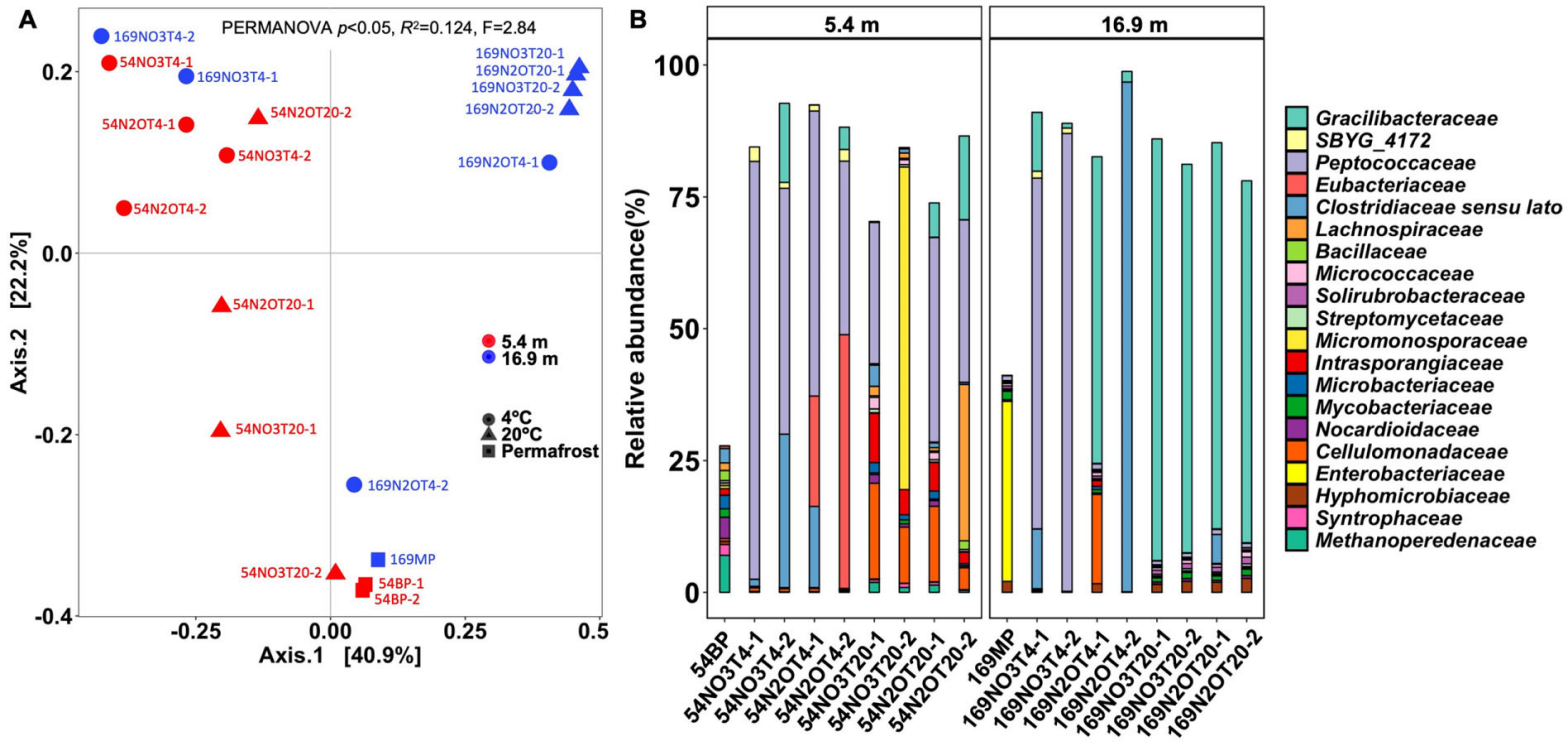
Microbial community composition of the pristine permafrost samples, and NO_3_^−^- and N_2_O-supplemented microcosms incubated at 4°C and 20°C based on 16S rRNA gene fragments recovered from the metagenomes.(A) Beta diversity of microbial communities based on weighted UniFrac analysis of 16S rRNA gene fragments recovered from the metagenomes. Relationships between samples were visualized by principal coordinates analysis (PCoA). The symbol colors distinguish sample depth; the symbol shapes indicate the pristine permafrost sample or microcosms incubated at 4°C and 20°C; the sample names indicate the source of nitrogen and the duplicate microcosms. (B) The relative abundance distributions of the top 20 families observed in the pristine permafrost samples and the different microcosms. The *x*-axis labels indicate individual metagenomes, while 54BP represent metagenome combined from replicates. The colors in the legend are ordered (top to bottom) to match the colors in the bar plot. Sample names contain reference to depth, source of nitrogen, incubation temperature (T4 means 4°C and T20 means 20°C), and replicate (detailed description of the microcosms’ naming is given in Supplementary Materials).

At the end of the incubation period, the microcosms were sacrificed for microbial community analysis. The major goal was to assess whether the enrichment conditions selected for different N_2_O-producing and N_2_O-consuming taxa over the 1-year incubation period, metagenome sequencing was performed on replicate microcosms within each treatment. The 16 metagenomic datasets generated from the individual microcosms (Table S2), as well as three datasets generated from the pristine permafrost (Table S3), revealed a total of 1,055 16S rRNA gene-based taxonomic assignments (Table S4). Beta diversity analysis, a measure of the dissimilarity between communities, indicated distinct community compositions in relation to the depth and temperature (PERMANOVA, model: dissimilarity ∼ depth + temperature; *P* < 0.05; *R*^2^ = 0.124; Fig. 2A). Based on 16S rRNA gene taxonomic assignments, the first two axes of the PCoA explained ∼63% of the variation in community dissimilarity (Fig. 2A and Table S5). Communities from pristine permafrost samples 54BP and 169MP group together, whereas incubation conditions (NO_3_^−^ versus N_2_O amendments and temperature) resulted in profound shifts in community composition (Fig. 2A). The modest *R*^2^ suggests that, in addition to depth and temperature, other factors and/or within-group variability contribute to overall community dissimilarity.

The majority of the 16S rRNA gene sequences from the 54BP permafrost were affiliated with four major phyla, *Actinomycetota, Bacillota, Pseudomonadota*, and *Methanobacteriota*, with a combined relative abundance exceeding 80% (Fig. S3). *Pseudomonadota, Chloroflexota, Actinomycetota*, and *Thermoproteota* sequences dominated the 169MP permafrost with a combined relative abundance of 87% (Fig. S3). In contrast, the majority of the NO_3_^−^- or N_2_O-supplemented microcosms at 4°C were dominated by sequences representing *Bacillota*, while microcosms at 20°C contained both *Bacillota* (15%–90%) and *Actinomycetota* (10%–18%) sequences (Fig. S3). The community analysis showed that sequences affiliated with *Peptococcaceae, Gracilibacteraceae*, and *Clostridiaceae* families of the phylum *Bacillota* increased in relative abundance in NO_3_^−^-supplemented microcosms using both 54BP and 169MP permafrost sediments at 4°C (Fig. 2B). In contrast, the microcosms 54NO3T20 (NO_3_^−^-supplemented 54BP samples incubated at 20°C) were dominated by *Cellulomonadaceae* (phylum *Actinomycetota*) and either *Peptococcaceae* (phylum *Bacillota*) or *Micromonosporaceae* (phylum *Actinomycetota*) sequences, while microbial communities in 169NO3T20 microcosms at similar conditions were dominated by *Gracilibacteraceae* (phylum *Bacillota*) sequences. In the N_2_O-supplemented microcosms incubated at 4°C, sequences representing *Peptococcaceae* and *Eubacteriaceae*, both in the phylum *Bacillota*, dominate the 54N2OT4 microcosms, whereas *Gracilibacteraceae* and *Clostridiaceae*, also members of the phylum *Bacillota*, dominate the 169N2OT4 microcosms. N_2_O-supplemented microcosms incubated at 20°C harbored the most diverse communities, as indicated by higher Shannon diversity (H = 2.50 in the 54BP series and H = 1.91 in the 169MP series) than the other microcosms (H ≤ 2.49 and H ≤ 1.67, respectively) (see Table S6 for alpha diversity indexes in each microcosm). Thus, the 54N2OT20 microcosms showed the presence of sequences affiliated with members of the *Gracilibacteraceae, Peptococcaceae*, and *Lachnospiraceae* families of the phylum *Bacillota* with sequences representing the family *Cellulomonadaceae* (phylum *Actinomycetota*) dominating the sequence pool, while sequences representing the family *Gracilibacteraceae* (phylum *Bacillota*) dominated the 169N2OT20 microcosms (Fig. 2B). Some members of these families (e.g. *Clostridiaceae, Peptococcaceae, Lachnospiraceae*) comprise species known to carry out DNRA, denitrification, and N_2_O reduction (Broman et al. 2021, Sun et al. 2024), suggesting that bacteria capable of using NO_3_^−^ and/or N_2_O as an electron acceptor(s) were enriched.

### Nitrogen cycling genes in assembled contigs

**Figure 3:**
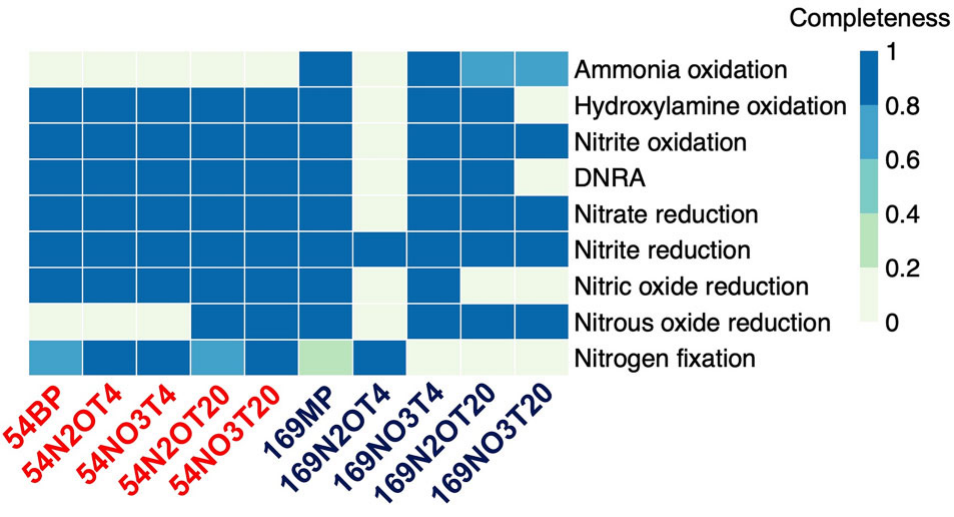
Functional analysis of assembled contigs derived from combined replicate metagenomes (except 169MP) of pristine permafrost samples and N_2_O- and NO_3_^−^-reducing microcosms. The heatmap shows the completeness of key metabolic pathways or functions implicated in nitrogen cycling based on KEGG annotation. Contigs recovered from 54BP and 169MP permafrost samples and corresponding microcosms are grouped by depth. Sample names contain reference to depth, source of nitrogen, and incubation temperature (T4 means 4°C and T20 means 20°C; detailed description of the microcosms’ naming is given in Supplementary Materials). The legend denotes the completeness of each metabolic pathway or function, where a value of 0 represents the absence of all associated genes, and a value of 1 indicates full completeness (100%).

The assembled sequence reads from two permafrost soils and their corresponding microcosms were thoroughly analyzed for functional prediction, with a focus on NO_3_^−^ reduction, denitrification, nitrification, and DNRA (Fig. 3). For the 5.4 m permafrost, only sequence assemblies from microcosms at 20°C showed the potential for complete denitrification (NO_3_^−^ to N_2_). Other assemblies from 5.4 m permafrost lack *nosZ* genes, consistent with the accumulation of N_2_O. For the 16.9 m permafrost, genes related to complete denitrification (i.e. from NO_3_^−^ → N_2_) were detected in the assemblies derived from the pristine 169MP sample and the NO_3_^−^-supplemented microcosms at 4°C (169NO3T4), while sequence assembly from 169N2OT4 microcosms lacked *nosZ* genes. Nitrification genes were detected in the assemblies from the pristine 169MP permafrost and microcosm 169NO3T4. Genes implicated in DNRA were not present in assemblies derived from microcosms 169N2OT4 and 169NO3T20. These findings suggest that available nitrogen sources and temperature affect the dynamics of populations implicated in nitrogen cycling in thawed Siberian permafrost.

### MAGs harboring denitrification and/or DNRA genes

**Figure 4:**
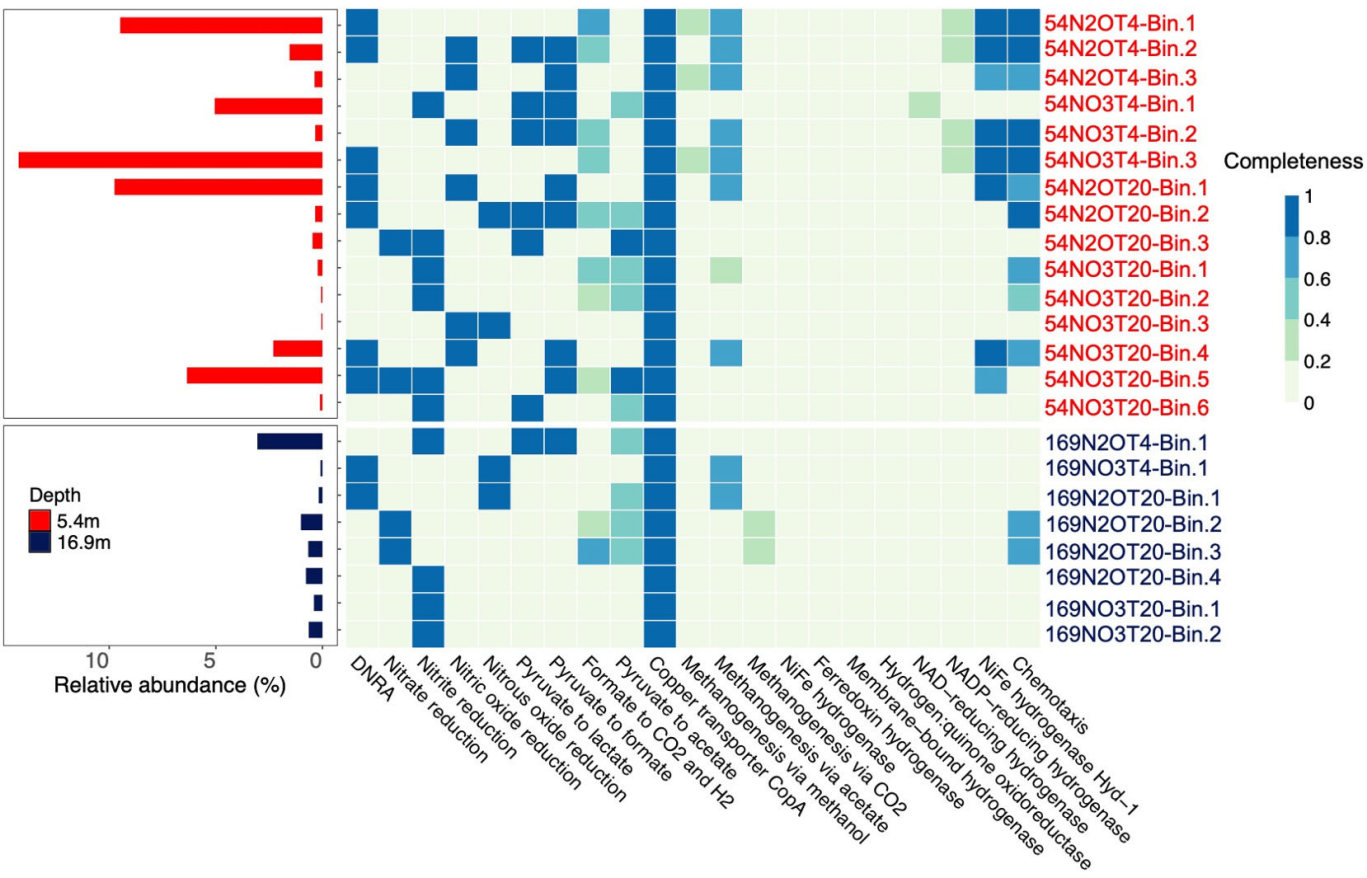
The relative abundance of MAGs harboring genes for denitrification and/or DNRA derived from the corresponding microcosms (left panel). Functional analysis of MAGs harboring nitrogen cycling genes (right panel). The heatmap shows the completeness of key metabolic pathways or functions in the 23 qualified MAGs harboring nitrogen cycling genes based on KEGG annotation. MAGs recovered from microcosms using permafrost from 5.4 m and 16.9 m are grouped by depth. The MAG names shown on the *x*-axis indicate the sample, substrate, and incubation temperatures (T4 means 4°C and T20 means 20°C; detailed description of the microcosms’ naming is given in Supplementary Materials). The legend denotes the completeness of each metabolic pathway or function, where a value of 0 represents the absence of all associated genes, and a value of 1 indicates full completeness (100%).

Among the 61 qualified MAGs recovered from the 16 metagenomes generated from the NO_3_^−^- and N_2_O-supplemented microcosms, 23 MAGs harbor genes predicted to be involved in denitrification and/or DNRA (Fig. 4, Tables S7 and S8). These 23 MAGs were assigned to five phyla, including *Actinomycetota* (9 MAGs), *Bacillota* (8 MAGs), *Pseudomonadota* (4 MAGs), and *Chloroflexota* (2 MAGs). Twenty of these 23 MAGs are assigned to novel species according to the ANI results at the 95% threshold level (Table S8), suggesting an unexplored microbial diversity related to N_2_O formation and consumption in ancient permafrost deposits. These 20 MAGs have relative abundances exceeding 0.1% in the corresponding metagenomes they were assembled from (Fig. 4). Most of these MAGs exhibit low or negligible relative abundances in the metagenomes derived from the pristine permafrost (Fig. S4), suggesting selective enrichment during the microcosm incubation.

### Functional analysis of MAGs harboring genes for denitrification and/or DNRA

Key metabolic pathways of the 23 MAGs harboring denitrification and/or DNRA genes were predicted based on KEGG annotation (Fig. 4). Canonical denitrification gene clusters to perform the entire denitrification process (i.e. NO_3_^−^→NO_2_^−^→NO→N_2_O→N_2_) were not detected in any of the recovered MAGs, and only one MAG (54NO3T20-Bin.9) encoded genes for DNRA (i.e. NO_3_^−^→NO_2_^−^→NH_4_^+^). Four MAGs harboring genes encoding NO_3_^−^ reductase (*narG* and/or *napA*) were identified in 20°C microcosms, one MAGs from each 54NO3T20 and 54N2OT20, and two MAGs from 169N2OT20 microcosms (Fig. 4). Ten MAGs harbor *nirK* (k00368) encoding the copper-dependent nitrite reductase for NO_2_^−^ reduction. *nirS* (k15864), encoding the cytochrome *cd_1_*-dependent nitrite reductase NirS, was not found. *norB* genes encoding nitric oxide reductase were identified in six MAGs derived from the 54NO3T20, 54N2OT20 and 54N2OT4 microcosms (Fig. 4). Genes for N_2_O reduction were identified in four MAGs, two of each derived from microcosms established using 54BP and 169MP permafrost samples. Three MAGs harbor genes involved in the process of denitrification, while no MAGs bear genes for more than two steps of the process. Specifically, two MAGs (54N2OT20-Bin.3 and 54NO3T20-Bin.5) contain genes for the reduction of NO_3_^−^ to NO, and one MAG (54NO3T20-Bin.3) harbors genes for the reduction of NO to N_2_. The genes for denitrification and/or DNRA were not found in unbinned contigs. These findings indicate that incomplete denitrifying bacteria may be the main drivers for N_2_O formation and non-denitrifying bacteria carry out N_2_O reduction in Siberian permafrost samples investigated.

In addition to the permafrost-associated organic substrates (Table S1), pyruvate served as the carbon source and source of reducing equivalents for the reduction of NO_3_^−^ and N_2_O. About 48% of microcosms’ MAGs harboring denitrification and/or DNRA genes also carry genes involved in metabolizing pyruvate to acetate, formate, and lactate (Fig. 4). These findings indicate that the majority of MAGs harboring denitrification and/or DNRA genes are capable of utilization of pyruvate as an electron donor. The last step of complete denitrification, the reduction of N_2_O to N_2_, is catalyzed by the well-characterized copper-containing enzyme NosZ and the expression of *nosZ* is partly controlled by the extracellular copper concentration (Sullivan et al. 2013). Detection of genes for copper transport in all microcosm-derived MAGs carrying denitrification and/or DNRA genes points that the corresponding organisms can take up copper from the environment.

### Phylogenetic distribution of *nosZ* genes derived from permafrost sediments and microcosms

**Figure 5:**
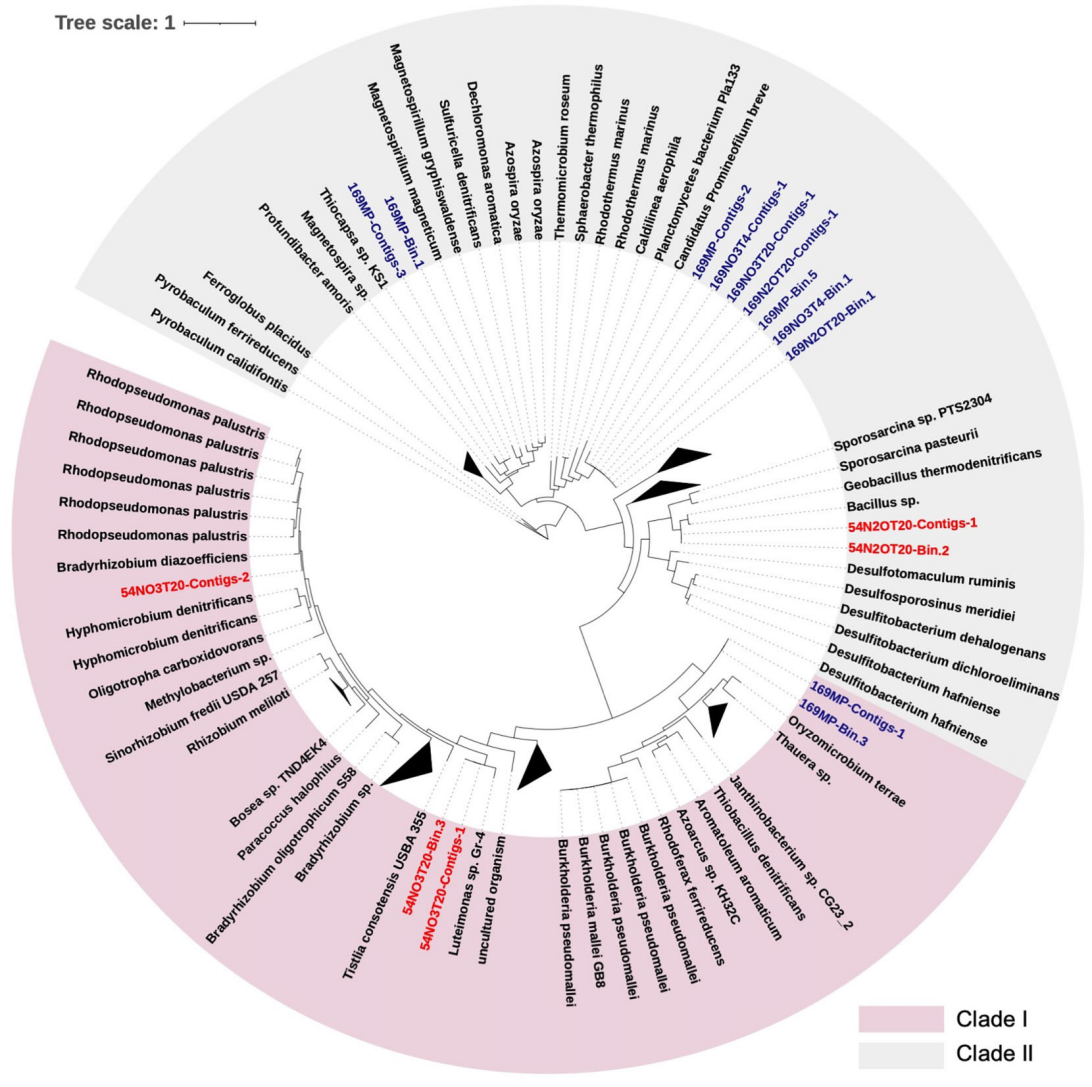
Phylogenetic diversity of the near full-length *nosZ* sequences recovered from assembled contigs (names contain word -Contigs), the qualified MAGs (names contain word -bin), and *nosZ* from the reference *nosZ* database. Branch labels indicate *nosZ* sequences derived from depths of 5.4 m and 16.9 m, respectively. The numbers 54 and 169 reflect the depth (5.4 m and 16.9 m) of permafrost. The letter and number strings N_2_O and NO_3_^−^ indicate microcosm amendment, but abbreviations BP and MP indicate pristine permafrost samples. T4 and T20 represent the incubation temperature of microcosms at 4°C and 20°C, respectively (detailed description of the microcosms’ naming is given in Supplementary Materials).

Nine near full-length *nosZ* genes were obtained from assembled contigs (designated with suffix Contigs in Fig. 5), including three Clade I *nosZ* genes and six Clade II *nosZ* genes. One Clade II *nosZ* gene was repeatedly found in both 169MP permafrost and subsequent microcosms’ metagenomes, but this *nosZ* gene was not detected in any metagenomes for the 54BP permafrost. However, three *nosZ* genes (two Clade II and one Clade I) were identified in the associated N_2_O-producing and N_2_O-consuming microcosms at 20°C only. Specifically, assembled contigs from microcosm 54NO3T20 showed the presence of two near full-length Clade I *nosZ* genes, and one of those genes was assigned to MAG 54NO3T20-Bin.3. The Clade II *nosZ* gene identified in assembled contigs of microcosm 54N2OT20 was binned into MAG 54N2OT20-Bin.2. All three *nosZ* genes identified in assembled contigs of the 169MP metagenome were binned into MAGs (Fig. 5 and Fig. S5). In the microcosms established with 169MP permafrost, three identical Clade II *nosZ* genes were found in assembled contigs from three microcosms incubated under the different conditions (169NO3T4, 169N2OT20, and 169NO3T20), and two of these genes were binned into MAGs (Figs. 4 and 5). Comparison of the *nosZ* genes identified in the pristine permafrost samples and the microcosms showed only one identical Clade II *nosZ* gene (169MP-Contigs-2), indicating the impact of the enrichment process on the community composition and the *nosZ* gene pool.

A phylogenetic analysis of 16 *nosZ* genes derived from the permafrost and the microcosms and 209 (121 Clade I and 88 Clade II) reference *nosZ* gene sequences revealed that about 67% of permafrost-derived *nosZ* genes affiliate with Clade II (Fig. 5). Three Clade II *nosZ* genes (Fig. 5), including two genes found in 169MP permafrost and 169MP-derived microcosms, affiliated with the genera *Thiocapsa* and ‘*Candidatus Promineifilum*’, and one *nosZ* gene from 54N2OT20 affiliated with the genus *Bacillus*. Three Clade I *nosZ* genes (Fig. 5), one gene from 169MP permafrost affiliated with the class *Betaproteobacteria* (e.g. genera *Burkholderia* and *Janthinobacterium*), and two *nosZ* genes identified in microcosm 54NO3T20 belonged to the phylum *Pseudomonadota*, one related to genera *Bradyrhizobium* or *Rhodopseudomonas*, and another affiliated with the genus *Luteimonas*. Taken together, these analyses suggest that *nosZ* genes from both clades contribute to N_2_O reduction and enrichment selects for specific N_2_O-consuming populations.

## Discussion

### Thawed permafrost microcosms reduce NO_3_^−^ to N_2_O

The results show that the permanently frozen Siberian Sea coastal permafrost harbor bacteria with the metabolic potential to reduce NO_3_^−^/NO_2_^−^ to N_2_O. The NO_3_^−^-supplemented microcosms accumulated N_2_O, demonstrating that permafrost microbiomes can reduce NO_3_^−^ to N_2_O and Siberian permafrost is a potential N_2_O source under warming scenarios. N_2_O consumption in microcosms was steady but slow and, in some microcosms, cumulative N_2_O consumption equaled or exceeded the net N_2_O formation (Fig. 1), depending on the incubation conditions. The findings are consistent with field N_2_O emission measurements and microcosm studies conducted with other types of permafrost, which collectively emphasize the potentially large contribution of thawing permafrost to greenhouse gas emissions and feedbacks to climate warming (Voigt et al. 2017, Yang et al. 2018, Marushchak et al. 2021, Yin et al. 2024). Considering the thawing of coastal permafrost promotes the decomposition of soil organic matter, replenishes NO_3_^−^/NO_2_^−^ pools, and fuels denitrification, such processes in anoxic permafrost deposits will likely enhance N_2_O formation and emissions (Voigt et al. 2017).

Incomplete denitrification has been recognized as a major source of N_2_O from thawed permafrost (Marushchak et al. 2011, Gil et al. 2017, Voigt et al. 2017, Yang et al. 2018). Our study demonstrates that the reduction of NO_3_^−^ in East Siberian Sea coast permafrost is incomplete and stalls at N_2_O as final product (Fig. 1A and B). However, slow but steady N_2_O consumption was observed in all N_2_O-supplemented permafrost microcosms (Fig. 1C and D), suggesting that permafrost microbiomes have the capacity to reduce N_2_O. Thawing permafrost and expansion of active layer soils undergoing seasonal temperature fluctuations are considered major N_2_O sources (Marushchak et al. 2021). The capacity for N_2_O reduction, as observed here, suggests that *in situ* N_2_O consumption may occur. N_2_O emissions therefore reflect an imbalance between N_2_O formation and consumption, raising questions about how long-term (decadal) environmental change will impact microbial activities and N_2_O fluxes from these coastal permafrost ecosystems.

### Incomplete denitrifiers responsible for N_2_O formation and consumption

Multiple known nitrogen cycling processes, including denitrification, nitrification, DNRA, and chemodenitrification, contribute to N_2_O formation in environmental systems (Hallin et al. 2018, Shan et al. 2021). Therefore, the N_2_O generated in the NO_3_^−^-supplemented permafrost microcosms may be derived from multiple processes. Comparative analysis of the MAGs derived from NO_3_^−^-supplemented microcosms revealed that genes for NO_3_^−^ reduction (*narG, napA*) were only present in MAGs affiliated with the family *Micromonosporaceae*, genes for NO_2_^−^ reduction (*nirS, nirK*) were mainly encoded on MAGs representing the families *Demequinaceae, Dermatophilaceae, Xanthobacteraceae*, and *Micromonosporaceae*, and the NO reductase gene (*norB*) is predominately carried by families of *Desulfitobacteriaceae* and *Hyphomicrobiaceae* (Fig. 4 and Table S8). Members of these families likely contribute to the reduction of NO_3_^−^ to NO_2_^−^ and to N_2_O (Figs. 1A, B, S2C, and S2D). Detailed analyses of MAGs obtained from 54BP and 169MP permafrost metagenomes in a previous study (Wu et al. 2023) did not identify any MAGs harboring the gene sets for denitrification (i.e. NO_3_^−^→NO_2_^−^→ NO→N_2_O→N_2_), which supports the findings of the current study that incomplete denitrifying bacteria drive N_2_O formation (Fig. S5 and Table S9). We considered the possibility of missing functional genes in the qualified MAGs. Because MAG inclusion was based on a ≥70% completeness threshold, the absence of specific genes in a MAG may reflect incomplete genome recovery (assembly/binning) rather than true biological absence. Nevertheless, the recovered qualified MAGs are generally high or medium quality, and genes that are not assembled/binned are often associated with genomic regions that are difficult to recover (e.g. repetitive or variable regions and mobile elements) and/or hypothetical genes (Meziti et al. 2021). The multi-step NO_3_^−^ reduction pathway is the main NO_3_^−^ reduction process in the ocean (Babbin et al. 2014, Sun et al. 2024), and may explain why denitrification genes are the dominant nitrogen cycling genes in East Siberian Sea coast permafrost. Another study showed that the DNRA and incomplete denitrification pathways dominated nitrogen cycling in long-term frozen permafrost (Dang et al. 2022). Overall, these findings suggest that multiple incomplete denitrifiers contribute to N_2_O formation in the East Siberian Sea coast permafrost microcosms.

Microbial N_2_O reduction is driven by bacteria harboring NosZ, which have been distinguished into Clade I and Clade II (Sanford et al. 2012, Jones et al. 2013). Phylogenetic analysis of the *nosZ* genes derived from microcosms showed that they affiliated with both Clade I and Clade II *nosZ* (Fig. 5), the latter typically encoded by non-denitrifiers (Sanford et al. 2012, Jones et al. 2013, Hallin et al. 2018, Shan et al. 2021). The Clade II *nosZ* genes dominated in the microcosm metagenomes and in qualified MAGs, suggesting that N_2_O reduction in the permafrost microcosms is mainly driven by incomplete denitrifying N_2_O reducers.

The microcosm study demonstrated NO_3_^−^ and N_2_O reduction activity in microcosms established with permafrost samples; however, replicate NO_3_^−^-supplemented microcosms established with homogenized permafrost performed inconsistently. A plausible explanation are with the existence of distinct microenvironments, and incomplete homogenization may lead to different initial microbiomes in replicate microcosms (Fig. 2) (Wu et al. 2023). Indeed, such variability is not uncommon in soil microcosm studies (Sun et al. 2024), although the reasons for this variability remain debated (Zhou and Ning 2017). Despite variability among replicate microcosms, the experimental results demonstrate that the enrichment conditions select for specific NO_3_^−^- and N_2_O-reducing taxa, identify mostly novel taxa involved in these processes, and demonstrate that distinct bacterial populations are involved in the formation and consumption of N_2_O in permafrost microcosms.

### Effects of permafrost type and microcosm incubation temperature on N_2_O formation

A striking performance difference in terms of NO_3_^−^ reduction was observed in microcosms established with 54BP and 169MP permafrost samples. NO_3_^−^ was effectively reduced in 54BP microcosms under both 4°C and 20°C temperature conditions, but NO_3_^−^ reduction was slow in 169MP microcosms irrespective of temperature. The difference in NO_3_^−^ reduction performance may be due to different metabolic capabilities of the respective permafrost microbiomes. Prior metagenome analysis has demonstrated that the 54BP permafrost contained genes encoding NO_3_^−^ to NO_2_^−^ metabolic function, but the 169MP permafrost contained additional metabolic potential including NO_3_^−^ to NH_4_^+^ reduction, denitrification, and sulfur reduction (Wu et al. 2023). Previous studies provided evidence that physicochemical properties, microscale heterogeneity, and permafrost age are key determinants shaping microbiome taxonomic diversity and microbial metabolic variability and evenness (Gilichinsky et al. 1995, Vorobyova et al. 1997, Abramov et al. 2021). The 16.9 m deep permafrost used in the current study formed ∼120 kyr ago and is ∼15 kyr older than the permafrost from the 5.4 m depth horizon (Abramov et al. 2021), has two-fold lower total carbon and nitrogen contents, and ∼five-fold higher salinity (Table S1) (Wu et al. 2023). Microbial abundance has been repeatedly reported to be negatively correlated with permafrost age and positively correlated with carbon content (Khlebnikova et al. 1990, Abramov et al. 2021). Therefore, the microbial community associated with deeper saline permafrost may have lost metabolic capabilities resulting in slow NO_3_^−^ reduction activity.

Another explanation for differences in NO_3_^−^ reduction activities observed in 54BP and 169MP microcosms is the difference in salinity of the permafrost. The salinity of the 169MP sample is 5.6 ppt (12 g l^−1^), about three times higher than that of 54BP. Previous studies have shown that increased salinity and total ionic strength impact microbial community composition resulting in lower NO_3_^−^ reduction and denitrification activities (Glass and Silverstein 1999, Mariangel et al. 2008, Huang et al. 2022), consistent with low NO_3_^−^ reduction observed in the 169MP microcosms.

Temperature also impacts denitrification activities. Upon thawing, permafrost microbial communities experience elevated temperatures, which may lead to rapid microbial community structure and metabolic changes of cold-adapted populations (Feller and Gerday 2003, Nikrad et al. 2016, Monteux et al. 2020), and results in higher NO_3_^−^ reduction activity. Our findings show a steady N_2_O formation in NO_3_^−^-supplemented 54BP and 169MP microcosms at 4°C, a temperature that reflects typical summer temperatures in high Arctic areas (Marushchak et al. 2021, Lacroix et al. 2022). Occasionally, the summer temperature in the permafrost active layer can reach 20°C (Rachlewicz and Szczuciński 2008, Marushchak et al. 2021, Lacroix et al. 2022), and our findings of N_2_O formation at this temperature suggest that microbiomes in some permafrost active layers can rapidly respond to such elevated warming.

### Implications

Based on current predictions, ∼50% of permafrost will be lost by 2100 (McGuire et al. 2018, Biskaborn et al. 2019, Pörtner et al. 2019), and substantial amounts of greenhouse gases will be emitted (Elberling et al. 2010, Voigt et al. 2017, 2020, Marushchak et al. 2021). The results of this microcosm study suggest that N_2_O formation exceeds N_2_O consumption under warming scenarios, and this imbalance can profoundly impact climate change outcomes. In fact, N_2_O formation was mainly driven by novel, yet-to-be-characterized, incomplete denitrifying bacterial taxa. Understanding the responses of permafrost microbiomes involved in nitrogen turnover, including N_2_O formation and consumption, is crucial for predicting N_2_O emissions and associated feedbacks. More broadly, nitrogen cycling can interact with carbon cycling and thereby influence the net emission balance of multiple greenhouse gases from thawing permafrost (Yin et al. 2024). This highlights that system-level understanding is essential to faithfully predict greenhouse gas emissions from thawing permafrost (Yin et al. 2024). In contrast to temperate ecosystems, taxonomic, physiological, and ecological information of the microorganisms controlling nitrogen cycling in permafrost soils is scarce. Further study of permafrost and active layer microbiomes controlling N_2_O fluxes is warranted to refine predicted outcomes under different warming scenarios, to develop (engineered) strategies for reducing N_2_O emissions by enhancing N_2_O consumption, and to achieve a more holistic understanding of greenhouse emissions from permafrost regions.

## Supplementary Material

fiag034_Supplemental_File

## Data Availability

All metagenomic data sets were deposited in the NCBI Short-Read Archive under project PRJNA925153, and their respective accession numbers are listed in Table S2. Scripts for bioinformatics pipeline and statistical analyses are available on GitHub.
