# Supplementary material for: Incomplete denitrifying bacteria drive N2O fluxes in ancient Siberian permafrost microcosms
Source: FEMS Microbiol Ecol. 2026 Mar 31;102(5):fiag034. doi: 10.1093/femsec/fiag034 (PMC13094547; doi:10.1093/femsec/fiag034)
Supplement: fiag034_Supplemental_File [file fiag034_supplemental_file.docx]

SUPPLEMENTARY MATERIALS

**Incomplete Denitrifying Bacteria Drive N_2_O Fluxes in Ancient Siberian Permafrost Microcosms**

Yanchen Sun^1#^, Xiaofen Wu^2§^, Oksana G. Zanina^3^, Elizaveta M. Rivkina^3^, Karen G. Lloyd^4†^, Frank E. Löffler^1,2,5,6*^, and Tatiana A. Vishnivetskaya^2,4*^

^1^Department of Civil and Environmental Engineering, University of Tennessee, Knoxville, Tennessee 37996, USA

^2^Center for Environmental Biotechnology, University of Tennessee, Knoxville, Tennessee 37996, USA

^3^Institute of Physicochemical and Biological Problems in Soil Science, Russian Academy of Sciences, Pushchino 142290, Russia

^4^Department of Microbiology, University of Tennessee, Knoxville, Tennessee 37996, USA

^5^Department of Biosystems Engineering and Soil Science, University of Tennessee, Knoxville, Tennessee 37996, USA

^6^Department of Biochemistry & Cellular and Molecular Biology, University of Tennessee, Knoxville, Tennessee 37996, USA

**Corresponding Authors**

*Tatiana A. Vishnivetskaya, University of Tennessee, Department of Microbiology, Ken and Blaire Mossman Building, 1311 Cumberland Avenue, Knoxville, TN 37996, USA. E-mail: [tvishniv@utk.edu](mailto:tvishniv@utk.edu)

*Frank E. Löffler, University of Tennessee, Department of Civil and Environmental Engineering, 325 John D. Tickle Building, 817 Neyland Drive, Knoxville, TN 37996, USA. E-mail: [frank.loeffler@utk.edu](mailto:frank.loeffler@utk.edu)

Present Addresses

^#^Department of Marine Chemistry and Geochemistry, Woods Hole Oceanographic Institution, Woods Hole, Massachusetts 02543, USA

^†^USC Dana and David Dornsife College of Letters, Arts and Sciences, University of Southern California, Los Angeles, California 90031, USA

SUPPORTING INFORMATION SUMMARY

Supplemental methods, 8 tables, 5 figures, and references.

**Key to names of samples, microcosms, and MAGs.**

1. Pristine permafrost samples are named starting with depth followed by condition:

54BP for 5.4 m brackish permafrost

169MP for 16.9 m marine permafrost

2. Microcosms are named starting with depth followed by condition, temperature, and replicate number 1 or 2:

54NO3T4 for 5.4 m NO_3_^-^-amended microcosm incubated at 4°C

54NO3T20 for 5.4 m NO_3_^-^-amended microcosm incubated at 20°C

54N2OT4 for 5.4 m N_2_O-amended microcosm incubated at 4°C

54N2OT20 for 5.4 m N_2_O-amended microcosm incubated at 20°C

169NO3T4 for 16.9 m NO_3_^-^-amended microcosm incubated at 4°C

169NO3T20 for 16.9 m NO_3_^-^-amended microcosm incubated at 20°C

169N2OT4 for 16.9 m N_2_O-amended microcosm incubated at 4°C

169N2OT20 for 16.9 m N_2_O-amended microcosm incubated at 20°C

3. The MAGs are named starting with depth followed by condition, temperature, and bin number.

For example, 54NO3T4-bin.11.

4. The Contigs, which were assembled from metagenome reads, are named starting with depth followed by condition, temperature, and contig number. For example, 54NO3T20-Contigs-1.

**Table S1.** Physicochemical properties of permafrost sediments (Wu *et al.*, 2023).

| Sample | 54BP | 169MP |
| --- | --- | --- |
| Depth (m) | 5.4 | 16.9 |
| Estimated age (kyr) | 100-105 | 105-120 |
| Temperature (°C) | -7.09 | -7.96 |
| pH | 7 | 7 |
| Total C^a^ (%) | 2.088 | 0.911 |
| Total N^a^ (%) | 0.158 | 0.077 |
| Methane, (µmol/kg) | 76 | 3 |
| Salinity (ppt) | 1.1 | 5.6 |
| CO_3_^2-^ (mmol/kg) | 2.5 | 2 |
| SO_4_^2-^ (mmol/kg) | 1.7 | 30.3 |
| Cl^-^ (mmol/kg) | 55 | 220 |
| Ca^2+^ (mmol/kg) | 18.5 | 22 |
| Mg^2+^ (mmol/kg) | 36 | 42 |
| K^+^ (mmol/kg) | 0.6 | 5.4 |
| Na^+^ (mmol/kg) | 33.2 | 170.6 |
| Total dissolved solids, mmol/kg | 147.5 | 492.3 |

^a^Total carbon and nitrogen are reported as % of dry weight soil; the remaining parameters measured are in mg/kg of soil at time of sampling (i.e., wet weight).

**Table S2.** The NCBI SRA accession numbers for 16 metagenomes generated in this study. Source of permafrost sediments and incubation conditions for individual microcosm are indicated.

|  |  |  | 54BP-1 | 54BP-2 | 169MP-1 | 169MP-2 |
| --- | --- | --- | --- | --- | --- | --- |
| 4°C | N_2_O | NCBI SRA No. | SRR23193551 | SRR23193550 | SRR23193561 | SRR23193560 |
|  | NO_3_^-^ | NCBI SRA No. | SRR23193563 | SRR23193562 | SRR23193559 | SRR23193558 |
| 20°C | N_2_O | NCBI SRA No. | SRR23193565 | SRR23193564 | SRR23193555 | SRR23193554 |
|  | NO_3_^-^ | NCBI SRA No. | SRR23193557 | SRR23193556 | SRR23193553 | SRR23193552 |

**Table S3.** Sample and sequence information of published metagenome data downloaded from the European Nucleotide Archive.

| **Sample**  **(Depth-Replica)** | **Metagenome ID*^a^*** | **Reference** |
| --- | --- | --- |
| 54BP-1 | SRR10907119 | (Wu *et al.*, 2023) |
| 54BP-2 | SRR10907120 |  |
| 169MP-1 | SRR10907113 |  |

***^a^***The metagenomic data sets were deposited in the European Nucleotide Archive (ENA) under project PRJNA601698. The sample 16.9-2 did not yield sequences of good quality (Wu *et al.*, 2023).

**Table S4.** Number of 16S rRNA gene-based taxonomic assignments derived from replicate samples for the pristine permafrost samples (one replicate for 16.9 m sediment) and replicate microcosms for each condition of N_2_O- and NO_3_^-^-amended microcosms.

| **Sample** | **Pristine permafrost samples** | | **N_2_O** | | | | **NO_3_^-^** | | | | |
| --- | --- | --- | --- | --- | --- | --- | --- | --- | --- | --- | --- |
| Temperature |  | | 4°C | | 20°C | | 4°C | | 20°C | | |
| Replicate | #1 | #2 | #1 | #2 | #1 | #2 | #1 | #2 | #1 | #2 |  |
| 54BP | 281 | 316 | 173 | 133 | 199 | 289 | 217 | 192 | 454 | 248 |  |
| 169MP | 593 | NA | 156 | 192 | 320 | 319 | 265 | 303 | 300 | 227 |  |

NA: The sample 16.9-2 did not yield sequences of good quality and the data from 16.9 m of pristine permafrost sample represents only one replicate.

**Table S5.** Results of statistical analysis examining the effects of depth and incubation time on microbial community composition from PERMANOVA analysis based on weighted-UniFrac distances.

| **Factor** | **Df** | ***R*^2^** | **F** | ***p*-value** |
| --- | --- | --- | --- | --- |
| Depth | 1 | 0.211 | 9.675 | 0.001 |
| Temperature | 2 | 0.315 | 7.229 | 0.001 |
| Depth × temperature | 2 | 0.124 | 2.836 | 0.018 |

**Table S6.** Alpha diversity of microbial communities with the number of 16S rRNA gene-based taxonomic assignments and Shannon diversity index.

| **MAG ID** | **Number of 16S rRNA gene-based taxonomic assignments** | **Shannon diversity index** |
| --- | --- | --- |
| 54N2OT4 | 153 | 1.72 |
| 54NO3T4 | 205 | 1.43 |
| 54N2OT20 | 244 | 2.50 |
| 54NO3T20 | 351 | 2.49 |
| 169N2OT4 | 174 | 1.45 |
| 169NO3T4 | 284 | 1.20 |
| 169N2OT20 | 320 | 1.91 |
| 169NO3T20 | 263 | 1.67 |

The numbers 54 and 169 reflect different depth (5.4 m and 16.9 m) of permafrost samples used to establish the microcosms.

**Table S7.** Summary statistics of the 23 qualified MAGs derived from N_2_O- and NO_3_^-^-amended microcosms harboring nitrogen-cycling genes.

| **MAG ID** | **Size (Mbp)** | **Completeness (%)** | **Contamination (%)** |
| --- | --- | --- | --- |
| 54N2OT4-Bin.1 | 4.59 | 98.02 | 0.85 |
| 54N2OT4-Bin.2 | 3.66 | 98.98 | 1.4 |
| 54N2OT4-Bin.3 | 3.68 | 98.17 | 3.43 |
| 54NO3T4-Bin.1 | 2.53 | 93.11 | 5.83 |
| 54NO3T4-Bin.2 | 3.88 | 91.65 | 3.29 |
| 54NO3T4-Bin.3 | 3.8 | 89.72 | 0.26 |
| 54N2OT20-Bin.1 | 3.42 | 99.36 | 1.7 |
| 54N2OT20-Bin.2 | 4.17 | 96.35 | 1.41 |
| 54N2OT20-Bin.3 | 3.05 | 73.96 | 4.87 |
| 54NO3T20-Bin.1 | 3.10 | 72.33 | 1.55 |
| 54NO3T20-Bin.2 | 2.78 | 71.51 | 3.48 |
| 54NO3T20-Bin.3 | 2.90 | 75.91 | 3.67 |
| 54NO3T20-Bin.4 | 3.55 | 99.36 | 1.7 |
| 54NO3T20-Bin.5 | 4.07 | 92.77 | 2.89 |
| 54NO3T20-Bin.6 | 2.14 | 74.53 | 1.94 |
| 169N2OT4-Bin.1 | 2.17 | 76.87 | 1.93 |
| 169NO3T4-Bin.1 | 3.14 | 72.78 | 3.63 |
| 169N2OT20-Bin.1 | 2.28 | 55.87 | 4.18 |
| 169N2OT20-Bin.2 | 1.93 | 76.95 | 9.9 |
| 169N2OT20-Bin.3 | 2.21 | 83.9 | 3.21 |
| 169N2OT20-Bin.4 | 2.05 | 95.11 | 0.86 |
| 169NO3T20-Bin.1 | 1.96 | 82.77 | 4.38 |
| 169NO3T20-Bin.2 | 2.01 | 89.57 | 1.15 |

The numbers 54 and 169 reflect different depth (5.4 m and 16.9 m) of permafrost samples used to establish the microcosms.

**Table S8.** GTDB-Tk taxonomic classification of the taxa represented by the 23 qualified MAGs with nitrogen-cycling genes derived from the metagenomes of 16 microcosms.

| **MAG ID** | **GTDB-Tk taxonomic classification** |
| --- | --- |
| 54N2OT4-Bin.1 | d__Bacteria;p__Bacillota;c__Desulfitobacteriia;o__Desulfitobacteriales;f__Desulfitobacteriaceae;g__Desulfosporosinus;s__ |
| 54N2OT4-Bin.2 | d__Bacteria;p__Bacillota;c__Desulfitobacteriia;o__Desulfitobacteriales;f__Desulfitobacteriaceae;g__;s__ |
| 54N2OT4-Bin.3 | d__Bacteria;p__Bacillota;c__Desulfitobacteriia;o__Desulfitobacteriales;f__Desulfitobacteriaceae;g__Desulfitobacterium_A;s__ |
| 54NO3T4-Bin.1 | d__Bacteria;p__Actinomycetota;c__Actinomycetes;o__Actinomycetales;f__Demequinaceae;g__Demequina;s__ |
| 54NO3T4-Bin.2 | d__Bacteria;p__Bacillota;c__Desulfitobacteriia;o__Desulfitobacteriales;f__Desulfitobacteriaceae;g__;s__ |
| 54NO3T4-Bin.3 | d__Bacteria;p__Bacillota;c__Desulfitobacteriia;o__Desulfitobacteriales;f__Desulfitobacteriaceae;g__Desulfosporosinus;s__ |
| 54N2OT20-Bin.1 | d__Bacteria;p__Bacillota;c__Desulfitobacteriia;o__Desulfitobacteriales;f__Desulfitobacteriaceae;g__Desulfitobacterium_A;s__ |
| 54N2OT20-Bin.2 | d__Bacteria;p__Bacillota;c__Bacilli;o__Bacillales_B;f__DSM-18226;g__Mesobacillus;s__Mesobacillus oceanisediminis_A |
| 54N2OT20-Bin.3 | d__Bacteria;p__Actinomycetota;c__Actinomycetes;o__Propionibacteriales;f__Propionibacteriaceae;g__Raineyella;s__ |
| 54NO3T20-Bin.1 | d__Bacteria;p__Actinomycetota;c__Actinomycetes;o__Actinomycetales;f__Dermatophilaceae;g__UBA4719;s__ |
| 54NO3T20-Bin.2 | d__Bacteria;p__Pseudomonadota;c__Alphaproteobacteria;o__Rhizobiales;f__Xanthobacteraceae;g__Pseudolabrys;s__ |
| 54NO3T20-Bin.3 | d__Bacteria;p__Pseudomonadota;c__Alphaproteobacteria;o__Rhizobiales;f__Hyphomicrobiaceae;g__Hyphomicrobium_C;s__ |
| 54NO3T20-Bin.4 | d__Bacteria;p__Bacillota;c__Desulfitobacteriia;o__Desulfitobacteriales;f__Desulfitobacteriaceae;g__Desulfitobacterium_A;s__ |
| 54NO3T20-Bin.5 | d__Bacteria;p__Actinomycetota;c__Actinomycetes;o__Mycobacteriales;f__Micromonosporaceae;g__Micromonospora;s__ |
| 54NO3T20-Bin.6 | d__Bacteria;p__Actinomycetota;c__Actinomycetes;o__Propionibacteriales;f__Propionibacteriaceae;g__Aestuariimicrobium;s__ |
| 169N2OT4-Bin.1 | d__Bacteria;p__Actinomycetota;c__Actinomycetes;o__Actinomycetales;f__Demequinaceae;g__Demequina;s__ |
| 169NO3T4-Bin.1 | d__Bacteria;p__Chloroflexota;c__Anaerolineae;o__Anaerolineales;f__UBA4823;g__PFL25;s__ |
| 169N2OT20-Bin.1 | d__Bacteria;p__Chloroflexota;c__Anaerolineae;o__Anaerolineales;f__UBA4823;g__PFL25;s__ |
| 169N2OT20-Bin.2 | d__Bacteria;p__Pseudomonadota;c__Alphaproteobacteria;o__Rhizobiales;f__Methyloligellaceae;g__Methyloceanibacter;s__ |
| 169N2OT20-Bin.3 | d__Bacteria;p__Pseudomonadota;c__Alphaproteobacteria;o__Rhizobiales;f__Methyloligellaceae;g__Methyloceanibacter;s__ |
| 169N2OT20-Bin.4 | d__Bacteria;p__Actinomycetota;c__Thermoleophilia;o__Solirubrobacterales;f__70-9;g__67-14;s__67-14 sp016649615 |
| 169NO3T20-Bin.1 | d__Bacteria;p__Actinomycetota;c__Thermoleophilia;o__Solirubrobacterales;f__70-9;g__67-14;s__ |
| 169NO3T20-Bin.2 | d__Bacteria;p__Actinomycetota;c__Thermoleophilia;o__Solirubrobacterales;f__70-9;g__67-14;s__67-14 sp016649615 |

The numbers 54 and 169 reflect different depth (5.4 m and 16.9 m) of permafrost samples used in the microcosm.

**Table S9.** GTDB-Tk taxonomic classification of the taxa represented by the 7 qualified MAGs with nitrogen-cycling genes derived from the metagenomes of pristine permafrost samples 54BP and 169MP.

| **MAG ID** | **GTDB-Tk taxonomic classification** |
| --- | --- |
| 54BP-Bin.1 | d__Bacteria;p__Actinomycetota;c__Actinomycetes;o__S36-B12;f__UBA10799;g__JADKAV01;s__ |
| 169MP-Bin.1 | d__Bacteria;p__Pseudomonadota;c__Gammaproteobacteria;o__UBA9214;f__UBA9214;g__UBA9214;s__ |
| 169MP-Bin.2 | d__Bacteria;p__Gemmatimonadota;c__Gemmatimonadetes;o__Palauibacterales;f__Palauibacteraceae;g__Carthagonibacter;s__Carthagonibacter sp016649455 |
| 169MP-Bin.3 | d__Bacteria;p__Myxococcota;c__UBA9160;o__UBA9160;f__SMWR01;g__CALDCK01;s__ |
| 169MP-Bin.4 | d__Bacteria;p__Desulfobacterota;c__Deferrimicrobia;o__Deferrimicrobiales;f__Deferrimicrobiaceae;g__CSP1-8;s__CSP1-8 sp030668225 |
| 169MP-Bin.5 | d__Bacteria;p__Chloroflexota;c__Anaerolineae;o__Anaerolineales;f__UBA4823;g__PFL25;s__ |
| 169MP-Bin.6 | d__Bacteria;p__Pseudomonadota;c__Gammaproteobacteria;o__Enterobacterales;f__Enterobacteriaceae;g__Enterobacter;s__Enterobacter cloacae |

**Figure S1.** Schematic of the study site and overview of the experimental design (see Materials and Methods for details). The metagenomes of the two pristine permafrost samples have been analyzed in a prior study (Wu *et al.*, 2023) and are available in the European Nucleotide Archive under project PRJNA601698. Each setup (defined as 54N2OT4, 54NO3T4, 54N2OT20, 54NO3T20, 169N2OT4, 169NO3T4, 169N2OT20, and 169NO3T20) consisted of two replicated microcosms. Additional replicates were not possible due to the limited amounts of permafrost sediments available. A total of sixteen microcosms amended with either NO_3_^-^ or N_2_O were set up. Negative controls included heat-killed (autoclaved) replicates and microcosms without N_2_O and NO_3_^-^ but with pyruvate at pH 7.2 for each permafrost sediment.

**Figure S2.** The NO_3_^-^ (upper) and NO_2_^-^ (low) profiles in the corresponding microcosms initially containing approximately 30 μmol NO_3_^-^ at 4°C (left) and 20°C (right), respectively.

**Figure S3.** Microbial community compositions at the phylum (top 20 phyla are shown) level based on 16S rRNA gene fragments recovered from metagenome data from pristine permafrost samples and the microcosms maintained at 4 and 20°C and with NO_3_^-^ and N_2_O.

**Figure S4.** The relative abundance of MAGs harboring nitrogen-cycling genes derived from microcosms in the corresponding pristine permafrost sediments based on metagenomic reads competitively mapped against the MAGs. MAGs recovered from 5.4 m and 16.9 m permafrost sediments are shown in red and blue font, respectively. Name of MAGs contain reference to depth, substrate, temperature, and bin number.

**Figure S5.** Functional analysis of 7 qualified MAGs harboring denitrification and/or DNRA genes derived from pristine permafrost samples. Left panel shows the relative abundance of each MAG in the corresponding microcosms. Right panel depicts a heatmap showing the completeness of key metabolic pathways or functions in the 7 qualified MAGs harboring denitrification and/or DNRA genes based on KEGG annotation. The legend denotes the completeness of each metabolic pathway or function, where a value of 0 represents the absence of all associated genes, and a value of 1 indicates full completeness (100%).

**References**

Wu X, Almatari AL, Cyr WA, Williams DE, Pfiffner SM, Rivkina EM, Lloyd KG & Vishnivetskaya TA (2023) Microbial life in 25-m-deep boreholes in ancient permafrost illuminated by metagenomics. *Environ Microbiomes* **18**: 33.
